# Clinical utility of skin biopsy detection of phosphorylated alpha-synuclein in an outpatient practice

**DOI:** 10.3389/fneur.2026.1868980

**Published:** 2026-08-25

**Authors:** Todd Levine, Roy Freeman, Nicole Murray, Alexandria Swanson, Colin T. Gibbons, Jason Crowell, Justin Phillips, Christopher H. Gibbons

**Affiliations:** 1 CND Life Sciences, Scottsdale, AZ, United States; 2 Beth Israel Deaconess Medical Center, Boston, MA, United States; 3 Wesleyan University, Middletown, CT, United States; 4 Norton Neurosciences, Louisville, KY, United States

**Keywords:** clinical utility, Parkinson’s, phosphorylated alpha-synuclein, skin biopsy, synucleinopathy

## Abstract

**Introduction:**

The neurodegenerative disorders characterized by deposition of phosphorylated alpha-synuclein (P-SYN) include Parkinson’s disease (PD), multiple system atrophy (MSA) and dementia with Lewy bodies (DLB). Diagnosis of synucleinopathies in clinical practice has been enhanced by recent updates to diagnostic guidelines. Despite these advances, many patients still fall between diagnostic guideline criteria creating challenges to clinical care.

**Methods:**

This was a retrospective chart review of patients seen in an outpatient private practice. The objective of this study was to determine the impact of skin biopsy detection of phosphorylated alpha-synuclein (P-SYN) in the clinical care of patients with movement or cognitive symptoms in whom a synucleinopathy was part of the differential diagnosis. The clinical diagnosis and treatment plan before, and after, skin biopsy detection for P-SYN was determined through detailed chart extraction, with ICD-10 codes used to define clinical diagnosis.

**Results:**

One hundred patients with suspected neurodegenerative disease were included in the chart review, 47 presenting with tremor, 18 with gait dysfunction, 11 with rigidity or bradykinesia and 24 with cognitive dysfunction. After skin biopsy testing for P-SYN, 60% of patients had a change in their ICD-10 documented diagnosis and 91% had a change to their clinical treatment plan.

**Conclusion:**

In patients with suspected neurodegenerative disease, skin biopsy detection of P-SYN supported changes in the diagnostic impression or management in the majority of patients in this study. These findings support the growing role of diagnostic biomarkers in the diagnosis and management of patients with suspected neurodegenerative synucleinopathies.

## 1 Introduction

The diagnosis of the synucleinopathies, Parkinson’s disease (PD), multiple system atrophy (MSA) and dementia with Lewy bodies (DLB), are based on established clinical criteria and supplemented with additional diagnostic testing (1–3). However, these criteria acknowledge that sensitivity and specificity in the diagnosis of these complex diseases is not optimal, particularly early on in the disease course (1, 2). Key elements of data that inform the differential diagnosis include duration of symptoms, response to treatment, and many primary and secondary symptoms and signs that evolve over years. In addition, experience and specialty of the treating physician plays a significant role in arriving at a correct diagnosis. Despite recent updates to diagnostic criteria, the accuracy of clinical diagnosis has not achieved concordance with autopsy gold standards (4, 5).

Immunofluorescent staining of skin to detect phosphorylated alpha-synuclein (P-SYN) is a recently developed, minimally invasive diagnostic biomarker with high sensitivity and specificity in patients with synucleinopathies (6–10). Cutaneous detection of P-SYN is playing a growing role in the clinical management of patients with neurodegenerative disease, necessitating studies to evaluate the clinical utility of testing (11). Three prior publications have reported the use of cutaneous P-SYN testing in in tertiary care academic practices (11–13). In all 3 studies, skin biopsy detection of P-SYN aided in both diagnosis and treatment in the majority of patients. The patients included in these studies had a wide range of clinical features including tremor, gait dysfunction, parkinsonism, ataxia, cognitive dysfunction and autonomic dysfunction with a differential diagnosis that included PD, DLB and MSA. In most cases, the presence or absence of P-SYN from a skin biopsy improved the diagnostic certainty in these disparate clinical presentations. However, tertiary care academic centers tend to specialize in more complex clinical cases that are not routinely seen in the general neurology practice setting. The goal of this study was to examine the role of skin biopsy detection of P-SYN in the diagnosis and treatment of patients in whom synucleinopathy was part of the differential diagnosis of patients seen in an outpatient private practice setting.

## 2 Methods

### 2.1 Study design

A retrospective chart review was performed on 50 consecutive male and female patients with a movement or cognitive disorder in whom a synucleinopathy was part of the differential diagnosis who completed a skin biopsy for detection of P-SYN between January 2022 and December 2024.

### 2.2 Standard protocol approvals, registrations, and patient consents

This study was approved as a retrospective chart review that did not require signed informed consent. The study was approved by Sterling IRB (#12218) on 8/16/2024.

### 2.3 Patient population

The patients were studied from a single large multi-specialty private practice neurology group from the Louisville Kentucky region of the United States that sees thousands of patients with neurological disease per year. The patients were seen clinically by one of two movement disorder specialists (J. C and J. P.) for a suspected neurodegenerative disease. The group of patients did not fall within standard diagnostic guidelines for any single disease (skin biopsy was only ordered in patients where a diagnostic question remained). Patients underwent skin biopsy by their treating neurologist when synucleinopathy (e.g., PD, MSA or DLB) was part of the differential diagnosis. During the study time-period, 50–120 skin biopsies were ordered per year for detection of P-SYN. Fifty consecutive male and 50 consecutive female patients with at least 12 months of clinical evaluation prior to skin biopsy and at least 6 months of clinical follow-up post skin biopsy were included in the study. Data extracted by chart review included demographic information, clinical history, physical examination findings, skin biopsy results, medications, referrals, imaging results, treatment decisions and changes to medications and diagnosis.

### 2.4 Diagnosis and treatment

Diagnosis prior to and after skin biopsy was extracted from ICD-10 codes and clinical notes. The ICD-10 code was used to define the diagnostic category, with specific details in the clinical notes to further refine the diagnosis based on physician documentation. Every effort was made to accurately reflect the diagnostic category reported by the treating physician. For example, a diagnosis of “parkinsonism” with a differential diagnosis that included Parkinson’s disease and drug induced parkinsonism was listed as “parkinsonism” to reflect the inherent diagnostic uncertainty. Changes to a diagnosis over time required additional information in the medical record. Changes to a clinical diagnosis were defined as a substantive change in suspected etiology (e.g., Parkinson’s disease changed to progressive supranuclear palsy and not just a different ICD-10 code for Parkinson’s disease), confirmation of diagnosis was defined as moving from a suspected disease to a more specific diagnosis (e.g., parkinsonism changed to Parkinson’s disease in the clinical record with appropriate documentation) and no change defined as staying with the same diagnosis (e.g., essential tremor remains essential tremor). In the setting of additional ancillary testing (such as neuroimaging, CSF or plasma biomarkers or neuropsychological testing) the results were included in the physician diagnosis at the time the test results were obtained. For example, if a DaTSCAN was obtained prior to skin biopsy, the result was included in the pre-biopsy diagnosis.

Changes to treatment were defined as initiating, modifying or stopping a medication based on language in the chart linking the decision to the skin biopsy results. For example, a patient with a diagnosis of “parkinsonism” that was on a moderate dose of carbidopa-levodopa with no clear benefit had a skin biopsy did not contain P-SYN, the physician changed the diagnosis to PSP and stopped the levodopa rather than continuing to titrate levodopa to a higher dose. Other changes in clinical care were captured when available in the medical record and included additional diagnostic testing, non-pharmacologic changes to treatment, referrals to other specialties or therapy.

### 2.5 Skin biopsy procedure

Standard 3-millimeter punch biopsies were obtained from posterior cervical region, the distal thigh and the distal leg after administration of local lidocaine (14). Skin samples were fixed in Zamboni paraformaldehyde solution and shipped for processing at a central laboratory (CND Life Sciences, Scottsdale AZ). After fixation, biopsies were washed and placed in a 20% glycerol cryoprotectant solution, frozen and sectioned into 50 micron thick samples. Skin was immunostained with antibodies to protein gene product 9.5 (PGP 9.5, anti-rabbit, Cederlane: Cat# CL95101), a pan-axonal marker, and phosphorylated alpha-synuclein (P-SYN: Wako: Cat# 015–25,191) (6, 7, 14–17). All biopsies underwent review by a certified pathologist to identify deposits of P-SYN that co-localized with nerve fibers stained by PGP 9.5.

### 2.6 Statistical analysis

Statistical analysis was performed using SPSS v18.0 (IBM, Chicago, IL). Group data are presented as mean ± standard deviation. Descriptive statistics are used throughout to present primary data. Binary skin biopsy results are reported (presence or absence of P-SYN). Tabular data are analyzed by chi-square or contingency table analysis of results. Sample size of 100 was estimated based on data from 2 prior studies showing similar effects with similar size studies (12, 13).

## 3 Results

One hundred patients who completed skin biopsies for the evaluation of synucleinopathy were included in the study (mean age 73.7 ± 8.0 years, range 52–87, 50 male, 50 female, demographic information listed in Table 1). Patients were evaluated for a primary movement disorder (76%) or a primary cognitive disorder (24%). A total of 30/50 male and 20/50 female patients had P-SYN detected on skin biopsy (12/24 P-SYN positive in the group presenting with cognitive complaints and 38/76 P-SYN positive in the group presenting with motor manifestations of disease). Medical records were available for an average of 26 months (range 12–130) prior to skin biopsy and 15 months (range 6–30) after skin biopsy.

**Table 1:** Demographic information and skin biopsy findings grouped by clinical presentation.

Clinical presentation	Age (mean ± SD)	*N*	Sex (M/F)	Skin biopsy with P-SYN (M/F)	Normal skin biopsy
Tremor	72.4 ± 6.9	47 (47%)	20/27	13/11	23
Gait dysfunction	74.4 ± 6.5	18 (18%)	8/10	4/3	11
Rigidity and/or bradykinesia	66.8 ± 8.1	11 (11%)	7/4	5/2	4
Cognitive dysfunction	79.3 ± 7.4	24 (24%)	15/9	8/4	12

Demographic overview of the findings by clinical presentation. Results presented as mean ± standard deviation (SD) or by percentage of total group.

### 3.1 Impact on diagnosis and treatment

Skin biopsy results (both positive and negative) supported changes in diagnosis in 60% of treated patients, with confirmation of a suspected diagnosis in an additional 20%. A total of 91% of patients had a documented change in their treatment plan in response to their skin biopsy. In total, 94% of patients (94 of 100) had either a change in their diagnosis and/or their treatment after receipt of the skin biopsy results.

### 3.2 Change in diagnosis

**Figure 1:**
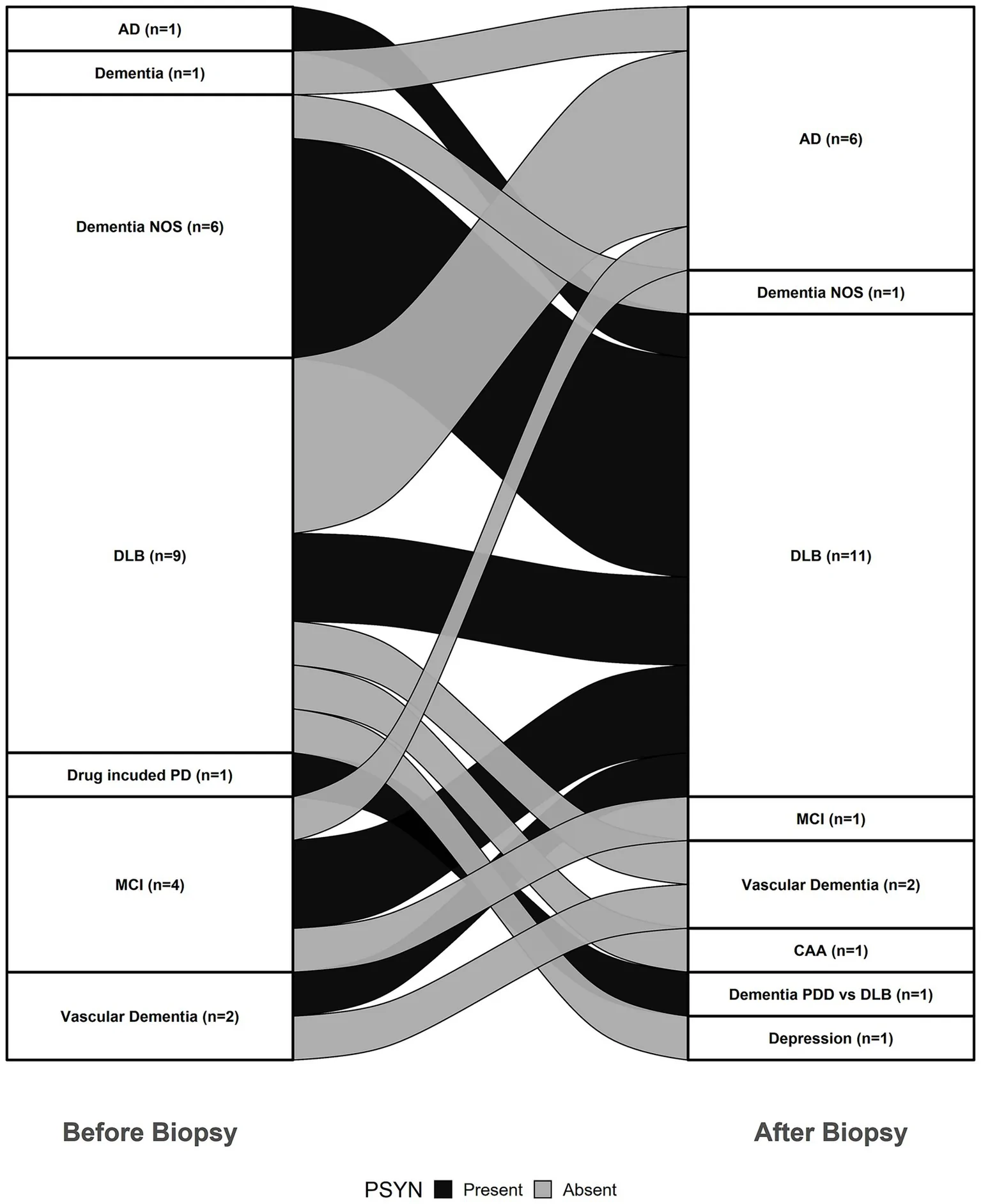
Alluvial plot depicting the pre-biopsy and post-biopsy diagnosis obtained from the medical records in patients presenting with cognitive dysfunction. Pre-biopsy diagnosis on the left, post-biopsy diagnosis on the right. Thickness of the flow indicates the number of patients that shift from the pre-biopsy diagnosis to the post-biopsy diagnosis. Grey flows are skin biopsies without P-SYN, black flow lines are skin biopsies with P-SYN.

**Figure 2:**
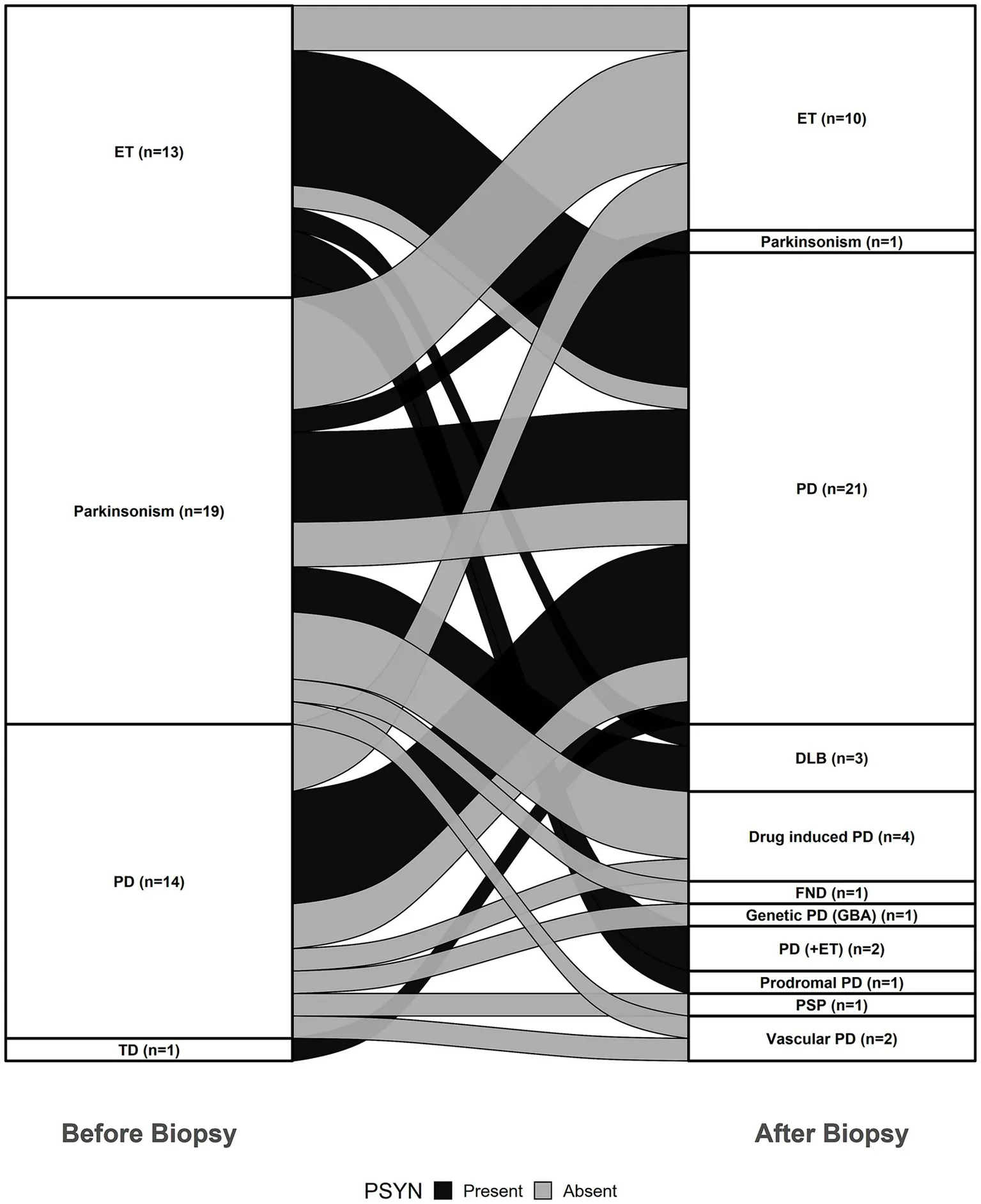
Alluvial plot depicting the pre-biopsy and post-biopsy diagnosis obtained from the medical records in patients presenting with tremor. Pre-biopsy diagnosis on the left, post-biopsy diagnosis on the right. Thickness of the flow indicates the number of patients that shift from the pre-biopsy diagnosis to the post-biopsy diagnosis. Grey flows are skin biopsies without P-SYN, black flow lines are skin biopsies with P-SYN.

**Figure 3:**
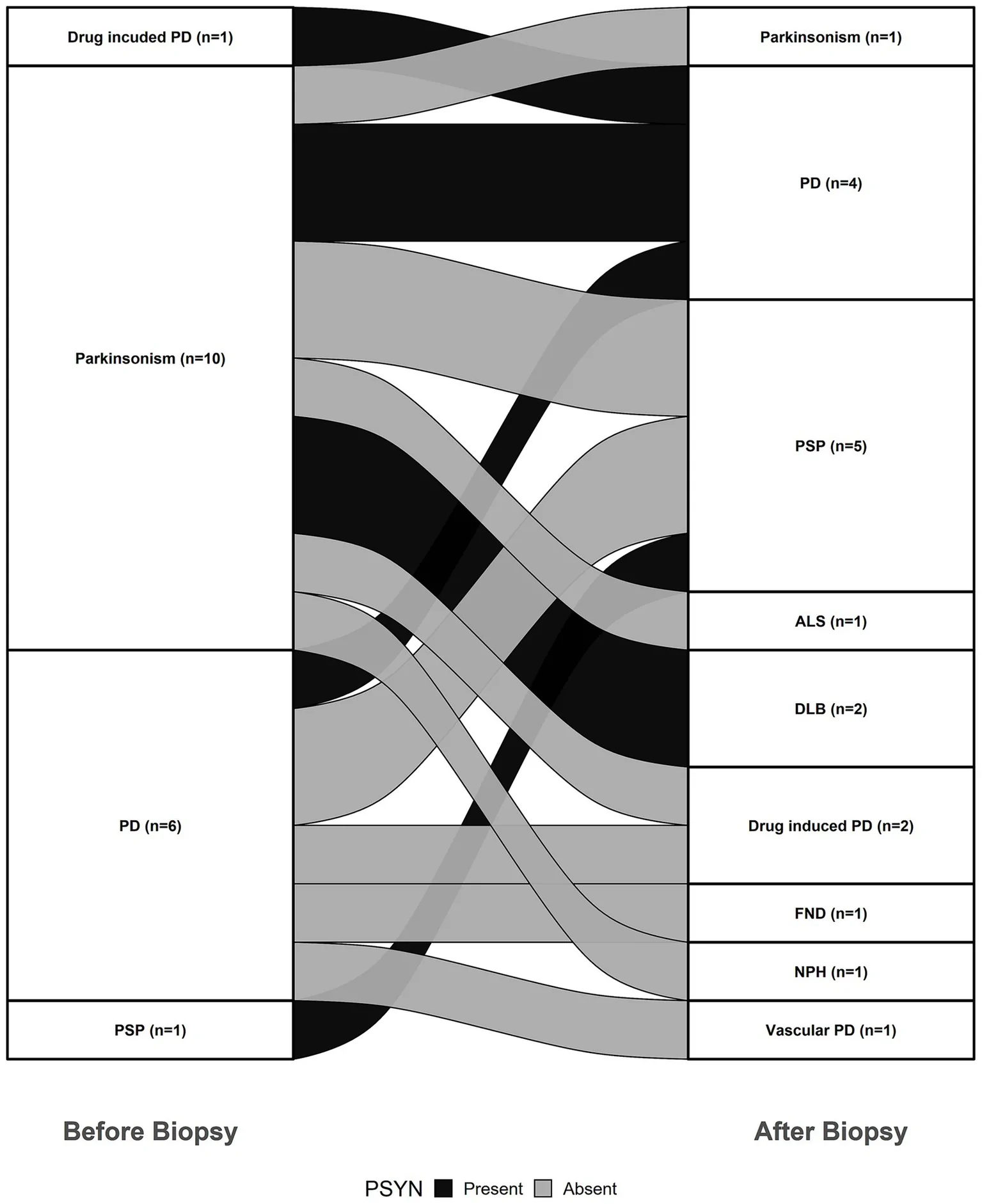
Alluvial plot depicting the pre-biopsy and post-biopsy diagnosis obtained from the medical records in patients presenting with gait dysfunction. Pre-biopsy diagnosis on the left, post-biopsy diagnosis on the right. Thickness of the flow indicates the number of patients that shift from the pre-biopsy diagnosis to the post-biopsy diagnosis. Grey flows are skin biopsies without P-SYN, black flow lines are skin biopsies with P-SYN.

**Figure 4:**
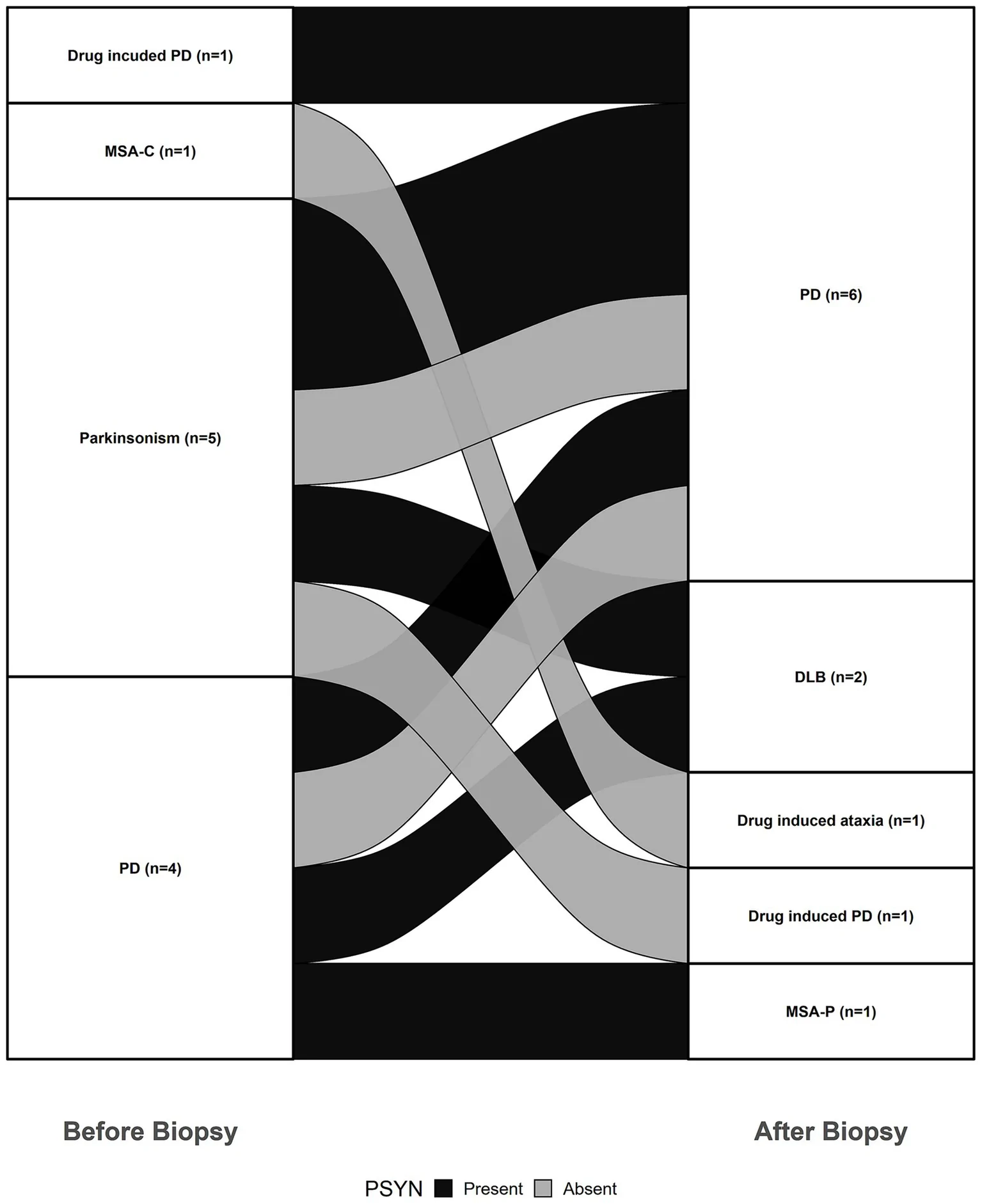
Alluvial plot depicting the pre-biopsy and post-biopsy diagnosis obtained from the medical records in patients presenting with rigidity and/or bradykinesia. Pre-biopsy diagnosis on the left, post-biopsy diagnosis on the right. Thickness of the flow indicates the number of patients that shift from the pre-biopsy diagnosis to the post-biopsy diagnosis. Grey flows are skin biopsies without P-SYN, black flow lines are skin biopsies with P-SYN.

A complete list of the clinical diagnoses before and after skin biopsy is shown in Figures 1–4, with data grouped by primary presenting symptom (cognitive dysfunction, tremor, gait instability or bradykinesia/rigidity). A skin biopsy was likely to result in a diagnosis change in both P-SYN positive and negative patients as shown in Figures 1–4.

*Cognitive Dysfunction*: The differential diagnosis of patients presenting with cognitive impairment included Lewy body dementia, Alzheimer’s disease, unspecified dementia, vascular dementia, and mild cognitive impairment. A change in diagnosis was documented in 21/24 (88%) of patients (Figure 1). The most frequent diagnosis change in P-SYN positive cases was from mild cognitive impairment and unspecified dementia to dementia with Lewy bodies in P-SYN positive cases (*n* = 5). In P-SYN negative cases, the most common change in diagnosis was dementia with Lewy bodies to Alzheimer’s disease (*n* = 4).

*Tremor*: Patients in whom tremor was the presenting symptom had a differential diagnosis that included essential tremor, drug-induced tremor and the tremors of Parkinson’s disease or parkinsonism. The skin biopsy was P-SYN positive in 51% (24/47) of patients with tremor, with a change in diagnosis in 75% (18/24) of the P-SYN positive cases, and 87% (21/23) of the P-SYN negative cases (Figure 2). The most common change in diagnosis in P-SYN positive cases was from essential tremor to Parkinson’s disease (*n* = 5). In P-SYN negative cases, the most common change in diagnosis was from Parkinson’s disease or parkinsonism to essential tremor (*n* = 8).

*Gait Instability*: Patients who presented with gait instability with suspected parkinsonism had a differential diagnosis that included Parkinson’s disease, drug included parkinsonism disease, parkinsonism or progressive supranuclear palsy. The skin biopsy was positive in 39% (7/18), with a change in recorded diagnosis in 71% (5/7) of positive cases, and 91% (10/11) of the negative cases (Figure 3). The most frequent diagnosis change in P-SYN positive cases was from drug induced parkinsonism to Parkinson’s disease (*n* = 2) and from drug induced parkinsonism to DLB (*n* = 2). In P-SYN negative cases, the most common change in diagnosis was Parkinson’s disease or parkinsonism changing to progressive supranuclear palsy (*n* = 4).

*Bradykinesia/Rigidity*: Patients in whom a skin biopsy was performed to evaluate parkinsonism with bradykinesia and/or rigidity had a differential diagnosis that included Parkinson’s disease, parkinsonism, drug included parkinsonism, progressive supranuclear palsy and multiple system atrophy. Skin biopsy was positive in 64% of cases (7/11), with a change in documented diagnosis in 86% (6/7) of positive cases, and 75% (3/4) of negative cases (Figure 4). The most frequent diagnosis change in P-SYN positive cases was from parkinsonism to Parkinson’s disease (*n* = 2). In P-SYN negative cases, the most common change in diagnosis was suspected parkinsonism changing to drug included parkinsonism (*n* = 1), and suspected multiple system atrophy to drug induced ataxia (*n* = 1).

### 3.3 Change in treatment

Skin biopsy supported changes in treatment in 91% of patients. Overall, a total of 143 medications were started, 66 medications were discontinued, and 48 medications had an adjustment in dose.

After biopsy results, treatment changes were as follows:

1. Dopamine replacement or dopamine agonists were started in 24 patients who were P-SYN positive with post-biopsy synucleinopathy diagnoses, and increased in 19 patients with post-synucleinopathy diagnosis who were not responding to lower doses of levodopa;
2. Levodopa was discontinued in 16 patients who were P-SYN negative and had a non-synucleinopathy post-biopsy diagnosis;
3. Cholinesterase inhibitors were started in 23 patients who were P-SYN negative with cognitive dysfunction;
4. Medications for essential tremor were started in 6 patients with post-biopsy essential tremor diagnoses without P-SYN on skin biopsy, and were discontinued in 4 patients in patients with P-SYN on biopsy;
5. Antipsychotic medications were started in 11 patients without P-SYN on skin biopsy and stopped on 6 patients with P-SYN on skin biopsy;
6. Antidepressant medications were started in 8 patients and discontinued in 8 patients after biopsy;
7. Treatment for orthostatic hypotension was started in 3 patients with P-SYN on skin biopsy;
8. After biopsy results, 41 new referrals were made to physical, occupational or speech therapy and 12 referrals were made to a multidisciplinary clinic;
9. Two patients without P-SYN on skin biopsy were referred for deep brain stimulation for control of tremor, with excellent clinical response.

## 4 Discussion

In this study, we report the use of skin biopsy detection of phosphorylated alpha-synuclein in complex neurodegenerative diseases in a private practice neurology specialty clinic. Although several recent studies highlight the utility of the P-SYN testing in tertiary care academic centers (11–13), to our knowledge this has not been previously described in an outpatient private practice setting. In the present study, the majority of patients underwent testing for either a cognitive or movement disorder symptom that did not meet clinical criteria for a specific underlying diagnosis leading to diagnostic uncertainty. Similar to prior publications (11–13), we found that the majority of patients had a change in documented diagnosis after cutaneous P-SYN testing, and over 90% had a recorded change in their treatment plan (12).

Clinical diagnostic sensitivity improves over time, with many studies reporting greatest accuracy after 5 years of clinical evaluation (4, 5, 18). However, in clinical practice it is not feasible to include a multi-year delay before providing a definitive diagnosis and plan of care. Diagnostic uncertainty is frustrating to patients and family members, and increases the risk of unnecessary treatments, testing and the potential for adverse reactions (19). The average duration of clinical evaluation and care in this cohort prior to skin biopsy was 26 months. An increase in diagnostic confidence was provided to 80% of the cohort after skin biopsy, possibly reducing the duration of diagnostic uncertainty by several years.

The patients seen in this study presented with a constellation of symptoms that we grouped into one of four categories including cognitive dysfunction, tremor, gait dysfunction or rigidity and/or bradykinesia. Due to the clinical heterogeneity in presentation, the physicians had a lower suspicion for synucleinopathy as a diagnosis compared to prospective clinical trials (6). Despite only 50% of the cases being positive for P-SYN, there was a recorded change in the underlying diagnosis in 60% of patients and a change in the underlying treatment in 91% of patients.

The most recent clinical diagnostic criteria for PD, MSA and DLB have greater sensitivity and specificity in making an accurate diagnosis, but have not yet incorporated synuclein biomarkers into their guidelines (1–3). Despite these advances, the diagnosis of synucleinopathies continues to be a major challenge, with accuracy often below 80%, particularly early in the disease course where overlapping symptoms include a broad differential diagnosis (4, 20, 21). As with the development of most new diagnostic technologies, periods of scientific debate occur in the literature prior to modifications to clinical guidelines (22). Although formal societal endorsement of a technology tends to be a later development, adoption of new tests in clinical practice can often highlight practical areas of clinical utility. In the present study, four discrete areas of diagnostic challenge occurred in our study cohort.

### 4.1 Tremor

Tremor is one of the most common neurologic symptoms, with over 1% of the population affected (23). Tremor was the most common presenting symptom in this cohort, with 47/100 patients reporting tremor as the reason for seeking medical care. Although the differential diagnosis for tremor is broad, with both degenerative and benign causes, physician and patient concern for a diagnosis of Parkinson’s disease is a major driver of clinical visits and evaluations (24). In this group, 79% (39/47) individuals had a change in diagnosis after skin biopsy, with equal change in the P-SYN positive and negative groups. The most frequently occurring overlapping diagnosis is essential tremor. In this study, 5 patients with suspected tremor-predominant PD had a negative skin biopsy and a recorded diagnosis change to essential tremor (Figure 2). This was accompanied by a change in treatment from dopaminergic therapy to a tremor targeted therapy. In addition, 7 patients with suspected essential tremor were found to have P-SYN on skin biopsy and had a recorded change in ICD-10 codes to Parkinson’s disease (Figure 2). Those 7 patients had changes in medical therapy that included the addition of dopaminergic treatment and the discontinuation of tremor specific therapy. These results suggest that skin biopsy detection of P-SYN was specifically helpful in aiding in a diagnosis in cases with mixed tremor phenotypes with overlapping PD and ET features. In this study, skin biopsy detection of P-SYN appeared to be performed in lieu of DaTScan, so direct comparison between results was not available.

### 4.2 Gait dysfunction

Gait dysfunction is a common cause for medical evaluation in aging patients and may be a major cause of morbidity (25). Primary gait dysfunction was the presenting symptom in 18 individuals in our cohort. The differential diagnosis of patients in this group is broad and includes Parkinson’s disease, vascular and other parkinsonism, multiple system atrophy, tauopathies (such as progressive supranuclear palsy and corticobasal syndrome) and non-neurologic causes of gait dysfunction (26). This group had the lowest rate of P-SYN positivity, with only 7/18 (39%) having P-SYN on skin biopsy (Figure 2). Of those positive cases, 6/7 were thought to have either idiopathic Parkinson’s disease or dementia with Lewy bodies and parkinsonism. One case was suspected to have PSP, and the physicians continued to suspect PSP despite a positive skin biopsy, although the possibility of co-pathology was raised.

#### 4.2.1 Case examples

An 83 year old male presented with gradual and progressive gait dysfunction including shuffling gait and decreased left arm swing. DaTScan did not suggest a dopaminergic deficit, so other conditions were considered. After emergence of a mild rest tremor, idiopathic PD was again considered, and skin biopsy was performed, and was negative for P-SYN. The patient was ultimately diagnosed with normal pressure hydrocephalus and neurosurgical shunt placement was performed, with clinical improvement over 12 months of follow up. Bladder and cognitive symptoms were not prominent in this case, making the diagnosis of NPH particularly challenging. The second case was a 76 year old male with gait dysfunction and bradykinesia that was initially thought to have undifferentiated parkinsonism, but did not respond to levodopa. Skin biopsy was P-SYN negative. Ongoing evaluation, including EMG, revealed findings suspicious for motor neuron disease and spasticity masquerading as bradykinesia. The patient was started on Riluzole for amyotrophic lateral sclerosis. Symptoms eventually became more widespread with bulbar involvement and the diagnosis of amyotrophic lateral sclerosis was clinically apparent. In both cases, a skin biopsy negative for P-SYN shifted the diagnostic pathway away from idiopathic PD, avoiding the potential for protracted levodopa trials and moved more rapidly to the final diagnosis, with major changes to treatment including shunt placement or disease modifying therapy.

### 4.3 Rigidity and/or bradykinesia

This was a smaller group of patients (*n* = 11) that presented primarily with rigidity and/or bradykinesia. This group had 64% P-SYN positivity (7/11). Given that rigidity and bradykinesia are part of the core clinical features of the diagnosis of PD (1), the higher rates of P-SYN positivity may not be a surprise. Overall, 8/11 were ultimately diagnosed with Lewy body disease (either DLB or PD). Two patients who developed mild cognitive impairment and later by bradykinesia were diagnosed with dementia with Lewy bodies (P-SYN positive), and 4/6 that were diagnosed with PD were P-SYN positive. There were two cases diagnosed with PD that were P-SYN negative:

1. The first P-SYN negative PD case had progressive rigidity and bradykinesia, but was not responsive to increasing doses of Sinemet. The primary diagnosis was idiopathic PD, but investigation into other parkinsonian syndromes was ongoing with progressive supranuclear palsy considered likely.
2. The second P-SYN negative case was diagnosed with idiopathic PD and had left sided parkinsonism that was responsive to levodopa. The diagnosis was complicated by both cervical myelopathy and a history of bipolar disorder that included use of antipsychotic medications leading to a differential diagnosis that included drug-induced parkinsonism.

There was one P-SYN positive patient diagnosed with multiple system atrophy based on the pattern of P-SYN deposition in the skin biopsy (7). The P-SYN deposition was topographically widespread, with a large total amount and frequent involvement of the subepidermal plexus (7).

### 4.4 Cognitive impairment

Cognitive impairment was the presenting symptom in 24 patients in this study. The differential diagnoses in these cases included Alzheimer’s disease, unspecified dementia, dementia with Lewy bodies, mild cognitive impairment, vascular dementia and drug included cognitive decline (Figure 2). Of the 24 patients, 50% were P-SYN positive, however, 75% of these patients had a change in diagnosis after skin biopsy. Only 2/9 individuals originally diagnosed with DLB had the same diagnosis post-biopsy. Similarly, of the 11 individuals with a final diagnosis of DLB, 9/11 were thought to have a non-synucleinopathy prior to biopsy. The prevalence of DLB is thought to be 3.6–6.6% of people older than age 65 (27). Despite the frequency of DLB in the older population, diagnostic accuracy, particularly in cases that do not present with other parkinsonian features, continues to be low (28).

The approval of anti-amyloid therapies in the treatment of Alzheimer’s disease has created a strong impetus to differentiate between disorders of synuclein and amyloid/tau proteins (29). Although there are high rates of co-pathology found at autopsy in both the AD and DLB population (30), the frequency with which synuclein is detected in the skin of patients with AD is still unknown, although longitudinal studies are currently underway.(16) Autopsy studies suggest that co-pathology has an adverse impact on the clinical course of disease, and therefore is an important factor to consider in the management of patients with cognitive decline (31, 32).

This study has a number of important limitations. First, this was a retrospective review of cases seen in a single private practice. Inherent to the chart review process is an interpretation of data, which can create the potential for a biased outcome. Despite efforts to utilize pre-specified criteria for diagnosis and treatment, this was not a blinded prospective trial and consensus criteria were not available to standardize the diagnosis. Furthermore, diagnosis was defined by the treating physician, not by an external gold standard. In addition, variable symptom types (such as tremor), were combined into a single clinical category despite major differences in clinical presentation (action, resting, mixed etc.) because of the small number of subjects. Second, this was a small study, and overinterpretation of data from small subgroups should be avoided. Sex specific differences between groups were not significantly different, but numbers were not powered to answer those questions. Third, skin biopsy detection of P-SYN does not provide a specific stand-alone diagnosis. Skin biopsy should be incorporated into physician decision making along with other clinical, laboratory and diagnostic information. Fourth, the issue of co-pathology, particularly in cognitive disorders, requires ongoing investigation. Fifth, changes to treatment (such as initiation of physical therapy or use of an anticholinesterase inhibitor) may overestimate the impact of the biopsy results because of temporal overlap in decision making. Sixth, routine use of other tests such as DaTSCAN or Alzheimer’s blood or CSF biomarkers were not available in this study. Lastly, the study incorporated data from a private practice and offered insights into diagnostic trends outside of academic medical centers but may not be generalizable to all populations.

Despite these limitations, this study reveals that skin biopsy detection of P-SYN, in combination with existing diagnostic tools and clinical data, supported a change in diagnostic impression or management of the majority of patients. These findings are similar to several similar recent studies of patients with suspected synucleinopathy treated at tertiary care medical centers, although rates of diagnosis and treatment changes were slightly higher in the present study (12, 13). Our data highlight the growing role of diagnostic biomarkers in the clinical care of patients with suspected synucleinopathies. Future studies are necessary to understand the relationship between different tests, such as DaTSCAN and skin biopsy, in the evaluation of patients with suspected synucleinopathies.

## Data Availability

The raw data supporting the conclusions of this article will be made available by the authors, without undue reservation.
